# Validation of tumor protein marker quantification by two independent automated immunofluorescence image analysis platforms

**DOI:** 10.1038/modpathol.2016.112

**Published:** 2016-06-17

**Authors:** Amy R Peck, Melanie A Girondo, Chengbao Liu, Albert J Kovatich, Jeffrey A Hooke, Craig D Shriver, Hai Hu, Edith P Mitchell, Boris Freydin, Terry Hyslop, Inna Chervoneva, Hallgeir Rui

**Affiliations:** 1 Department of Pathology, Medical College of Wisconsin, Milwaukee, WI, USA; 2 John P. Murtha Cancer Center, Walter Reed National Military Medical Center, Bethesda, MD, USA; 3 Chan Soon-Shiong Institute of Molecular Medicine at Windber, Windber, PA, USA; 4 Department of Medical Oncology, Thomas Jefferson University, Philadelphia, PA, USA; 5 Division of Biostatistics, Thomas Jefferson University, Philadelphia, PA, USA; 6 Duke Cancer Institute, Department of Biostatistics and Bioinformatics, Duke University, Durham, NC, USA

## Abstract

Protein marker levels in formalin-fixed, paraffin-embedded tissue sections traditionally have been assayed by chromogenic immunohistochemistry and evaluated visually by pathologists. Pathologist scoring of chromogen staining intensity is subjective and generates low-resolution ordinal or nominal data rather than continuous data. Emerging digital pathology platforms now allow quantification of chromogen or fluorescence signals by computer-assisted image analysis, providing continuous immunohistochemistry values. Fluorescence immunohistochemistry offers greater dynamic signal range than chromogen immunohistochemistry, and combined with image analysis holds the promise of enhanced sensitivity and analytic resolution, and consequently more robust quantification. However, commercial fluorescence scanners and image analysis software differ in features and capabilities, and claims of objective quantitative immunohistochemistry are difficult to validate as pathologist scoring is subjective and there is no accepted gold standard. Here we provide the first side-by-side validation of two technologically distinct commercial fluorescence immunohistochemistry analysis platforms. We document highly consistent results by (1) concordance analysis of fluorescence immunohistochemistry values and (2) agreement in outcome predictions both for objective, data-driven cutpoint dichotomization with Kaplan–Meier analyses or employment of continuous marker values to compute receiver-operating curves. The two platforms examined rely on distinct fluorescence immunohistochemistry imaging hardware, microscopy *vs* line scanning, and functionally distinct image analysis software. Fluorescence immunohistochemistry values for nuclear-localized and tyrosine-phosphorylated Stat5a/b computed by each platform on a cohort of 323 breast cancer cases revealed high concordance after linear calibration, a finding confirmed on an independent 382 case cohort, with concordance correlation coefficients >0.98. Data-driven optimal cutpoints for outcome prediction by either platform were reciprocally applicable to the data derived by the alternate platform, identifying patients with low Nuc-pYStat5 at ~3.5-fold increased risk of disease progression. Our analyses identified two highly concordant fluorescence immunohistochemistry platforms that may serve as benchmarks for testing of other platforms, and low interoperator variability supports the implementation of objective tumor marker quantification in pathology laboratories.

Analysis of protein markers in histological sections of formalin-fixed, paraffin-embedded tumors using brightfield microscopy and diaminobenzidine chromogen immunohistochemistry is widely used in pathology laboratories. Chromogen immunohistochemistry is being used to select oncology treatment regimens and for research to identify new prognostic and predictive biomarkers. For instance, chromogen immunohistochemistry has been widely used over the past two decades to detect protein expression of estrogen receptors, progesterone receptors, and Her2 in breast cancer and guide clinical management. However, many other promising chromogen immunohistochemistry biomarkers have failed to be implemented into clinical practice, in part, because of limitations of visual *in situ* immunoscoring. Currently, pathologists subjectively evaluate tumor marker levels based on chromogen immunohistochemistry staining intensity. This appraisal provides discrete, discontinuous data in the form of either ordinal (eg, low, medium, and high) or nominal (positive/negative) scores. These discrete scores are qualitative and not quantitative, and further suffer from inter- and intraobserver variability.^1, 2, 3, 4, 5, 6^ Limitations of pathologist-assessed chromogen immunohistochemistry scoring include subjectivity, poor resolution of crude discontinuous scoring metrics, and restricted dynamic range of chromogen signal intensity. The human eye has limited ability to accurately capture intensity differences, particularly at the upper and lower ends of detection and is susceptible to visual contrast illusions.^2, 3^

A number of digital pathology platforms now overcome the subjectivity of visual assessment and allow quantification of chromogen or fluorescence signals by computer-assisted image analysis, providing continuous immunohistochemistry values. These computer-assisted imaging platforms rely on histology image segmentation and feature extraction-based signal quantification algorithms to measure the signal intensity within tissue regions, cells, or subcellular compartments.^7, 8, 9^ Some of these platforms measure chromogen immunohistochemistry-stained slides and have received FDA approval for clinical use in breast cancer, including Ariol (Genetix/Leica Biosystems), Genie (Aperio Technologies/Leica Biosystems), and VIAS (Ventana Medical Systems).^7, 8, 9^ Other platforms use multiplexed fluorescence immunohistochemistry to measure targets within tissue regions or subcellular compartments defined by molecular colocalization of specific markers to derive automated region-specific intensity scores, including AQUA (HistoRx/Genoptix), Tissue Studio (Definiens), inForm (Caliper/Perkin-Elmer), MultiOmyx (Clarient), StrataQuest/TissueQuest (TissueGnostics), and BIOtopix/ONCOtopix (Visopharm).^7, 8, 9^

Immunofluorescence-based imaging of protein expression in formalin-fixed, paraffin-embedded tissue sections represents a superior alternative to chromogen-based *in situ* biomarker quantification owing to greater dynamic signal range and enhanced opportunities for multiplexed staining. The functional dynamic range of signals for fluorescence immunohistochemistry is 2–2.5 orders of magnitude, whereas chromogen immunohistochemistry has a dynamic range of only one order of magnitude.^7^ Higher sensitivity by immunofluorescence signals further allows for detection of biomarkers at reduced antibody concentrations or reduced incubation times, thus reducing nonspecific staining. Quantitative fluorescence immunohistochemistry analysis with unbiased image analysis solutions and signal intensity values as continuous variables permit the identification of sub-populations of immunolabeled cells that are not discernable by the human eye, as reported for biomarkers such as *β*-catenin and Her2 for breast cancer^2, 10, 11^ and AMACR for prostate cancer.^12^

Despite rapid developments in machine-based readers and software for multicolor fluorescence-stained histological slides over the past 10–15 years, commercially available slide scanners and image software solutions differ in features and capabilities. Owing to the differing features, it is difficult to validate claims of objective quantitative fluorescence immunohistochemistry data, especially if benchmarked against chromogen immunohistochemistry assays, which have more limited signal range, whether chromogen immunohistochemistry is analyzed by subjective and variable pathologist scoring or by a machine-based image analysis application. A single study applied two different quantitative platforms, the fluorescence-based AQUA platform (HistoRx/Genoptix) and the chromogen-based Ariol platform (Genetix), to validate the 'IHC4 multiparameter marker' algorithm on the same breast cancer cohort.^13^ Although both platforms validated the algorithm, the computed IHC4 scores were discordant and indicated greater analytical sensitivity on the fluorescence-based AQUA platform over the chromogen-based Ariol platform.^13^ However, no study has validated two fluorescence immunohistochemistry platforms against each other. In the absence of an established gold standard for quantitative immunohistochemistry data, if two different multicolor immunofluorescence analysis platforms both support manufacturers' claims of providing objective and quantitative immunohistochemistry data, the immunohistochemistry values for prognostic tumor markers should be highly concordant and yield comparable clinical outcome predictions. We therefore evaluated two technologically distinct commercial fluorescence-capable immunohistochemistry quantification platforms, the PM2000/AQUA platform (HistoRx/Genoptix) and the ScanScopeFL (Aperio/Leica Biosystems)/TissueStudio (Definiens) platform, using linear calibration to adjust for platform-specific parameters.

We previously have computed fluorescence immunohistochemistry data for multiple protein biomarkers using the PM2000 imaging hardware (HistoRx/Genoptix) and the AQUA imaging software (HistoRx/Genoptix).^14, 15, 16, 17, 18, 19, 20^ The PM2000 is an automated-stage immunofluorescence microscope that merges multichannel still images across the entire histological section. Using thresholds set in the pancytokeratin signal channel to identify epithelial/cancer cell regions and the DAPI signal channel to identify cell nuclei, AQUA generates tissue, cytokeratin, and nuclear compartments. Mean pixel intensity of the target biomarker channel is then calculated within a compartment of interest. An alternative fluorescence immunohistochemistry platform combines the ScanScopeFL (Aperio/Leica Biosystems) for image capture with image analysis by Tissue Studio (Definiens).^19, 20, 21^ The ScanScopeFL is a line scanner that captures an entire histological slide as a single, high-resolution, multichannel image. Tissue Studio relies on machine learning of user-guided representative tissue areas to generate an analysis solution that defines specific regions. Regions are defined globally as regions of interest such as cancer or stroma, as well as local spatial resolution at the cellular level with the identification of cell nuclei and cellular boundaries. The algorithm-based analysis solution is then applied to the entire slide to obtain multiparametric data within designated regions of interest.

For the present validation studies, we analyzed levels of nuclear-localized and tyrosine-phosphorylated Stat5a/b (Nuc-pYStat5) in breast cancer tissue microarray cohorts. We provide novel data revealing consistent and highly concordant Nuc-pYStat5 levels after linear calibration between the two distinct fluorescence immunohistochemistry platforms. Objective, data-driven cutpoint determination of quantitative Nuc-pYStat5 values derived by either of the two distinct fluorescence immunohistochemistry platforms independently identified comparable subgroups of breast cancer patients at elevated risk of disease recurrence. Importantly, data-driven cutpoints established on either platform could be successfully transformed into a correspondingly effective cutpoint for the values derived by the other platform. The new outcome data were furthermore consistent with our previously reported increased risk of failure of antiestrogen therapy in a subgroup of breast cancer patients with low levels of the Nuc-pYStat5 biomarker as detected by fluorescence immunohistochemistry using the PM2000/AQUA platform.^17^

## Materials and methods

### Paraffin-Embedded Breast Tumor Tissues

Breast cancer tissue microarrays were constructed from formalin-fixed, paraffin-embedded tumor specimens from Thomas Jefferson University Hospital pathology archives obtained under IRB-approved protocols. Cohort 1 represented 323 unselected cases of invasive breast cancer. The subset of 193 estrogen receptor-positive patients from Cohort 1 with nuclear-localized pYStat5a/b (Nuc-pYStat5) data for all immunohistochemistry assays was used in outcome analyses. These patients were diagnosed between the years 1995 and 2000. Clinical follow-up data ranged from 1 to 205 months. Cohort 2 represented 382 non-overlapping cases of estrogen receptor-positive breast cancer.

### Chromogen Immunohistochemistry and Scoring

Chromogen immunohistochemistry for Nuc-pYStat5 was performed on an Autostainer Plus (Dako) in a CLIA-certified laboratory using a previously described protocol.^17, 22^ Nuc-pYStat5 was reviewed by a board-certified breast cancer pathologist (JAH) and percent positively stained cancer cells were estimated, with detectable staining ranging from 1% to 95% in the cohort examined.

### Quantitative Immunofluorescence

Immunofluorescent staining of pYStat5 was performed on an Autostainer Plus (Dako) as described previously.^17^ For the PM2000/AQUA fluorescence immunohistochemistry platform (PM2000/AQUA), an image capture location was placed in the center of each core of the tissue microarray. The slide was automatically scanned using the PM2000 hardware (HistoRx/Genoptix) and fluorescent images were captured at x20 in three channels, DAPI (cell nuclei), fluorescein isothiocyanate/Alexa-488 (cytokeratin), and Cy5 (pYStat5) at the designated spots. AQUA (HistoRx/Genoptix) scores for Nuc-pYStat5 were calculated for each tumor as mean signal intensity within the cancer cell nuclei based on the epithelial compartment as defined by pancytokeratin- and DAPI-positive mapping. For the ScanScopeFL/TissueStudio fluorescence immunohistochemistry platform (ScanScopeFL/TissueStudio), a digital image of each channel (DAPI, fluorescein isothiocyanate/Alexa-488, and Cy5) on the entire slide was captured at x20 using the ScanScopeFL (Aperio/Leica Biosystems). Quantitative analyses were performed using the Tissue Studio (Definiens) digital pathology image analysis software. User-guided machine learning was performed on representative tissue areas to generate an analysis solution that defines specific regions of interest (eg, cancer, stroma). Detection of nuclei and cancer cells were facilitated by DAPI and pancytokeratin staining intensities and size thresholds. The analysis solution was then applied to the entire slide of tissue microarray Cohort 1 specimens and Nuc-pYStat5 scores were computed for each tumor as mean signal intensity within the cancer cell nuclei. The process was repeated on tissue microarray Cohort 2, which was independently stained and scanned 2 months after tissue microarray Cohort 1.

### Statistical Methods

The agreement between the log-transformed immunohistochemistry values obtained using PM2000/AQUA and ScanScopeFL/TissueStudio fluorescence immunohistochemistry platforms was evaluated using the extension of the Bland–Altman assay comparison method^23^ described by Carstensen^24^ and implemented in R package ‘MethComp'.^25^ The underlying model assumes that measurements by each of the two assays (PM2000/AQUA and ScanScopeFL/TissueStudio fluorescence immunohistochemistry platforms) are related linearly to the unknown ‘true' values of fluorescence immunohistochemistry signal with additional errors independent for the two assays. This linear relationship also implies a linear relationship between the predicted values for the two assays and between the predicted differences and averages of the two assays.^24^ The regression line for linear relationship between differences and averages of the two assays was obtained^23^ and the resulting regression parameter estimates were used to obtain the conversion equations for linear calibration between the two assays (coefficients of the linear relationship between the predicted values for the two assays) and the corresponding prediction intervals between assays.^24^ The concordance correlation coefficient with the corresponding 95% confidence intervals^26^ were computed as a measure of agreement between linearly calibrated PM2000/AQUA values and original ScanScopeFL/TissueStudio values, and *vice versa*. Recurrence-free survival was calculated in months from the date of diagnosis to date of first recurrence where there was a recurrence and equaled date of last contact or death in the absence of breast cancer recurrence. Recurrence-free survival was analyzed using the Kaplan–Meier survival curve estimator, log-rank test, and Cox proportional hazards model with dichotomized expression of Nuc-pYStat5 as a predictor. The fluorescence immunohistochemistry values of Nuc-pYStat5 from the two immunohistochemistry platforms were also analyzed as continuous predictors of recurrence-free survival using time-dependent receiver-operating curves.^27, 28^ As outcome is time-dependent, receiver-operating curves were produced for recurrence-free survival and compared in terms of area under the receiver-operating curve. Notably, calibration of the fluorescence immunohistochemistry marker values between platforms was not necessary for the receiver-operating curve analyses. For chromogen immunohistochemistry, optimal cutpoint for negative *vs* positive expression was determined to be the lowest level of detectable Nuc-pYStat5 recorded by the pathologist review, representing 1% positively stained cells. Recursive partitioning with 10 cross-validations was used in R package ‘rpart'^29^ to establish data-driven optimal cutpoints for dichotomization (high *vs* low) of fluorescence immunohistochemistry levels. Statistical analyses were performed in R.^30^

## Results

### Quantitative Immunofluorescence Data Derived on Two Independent Fluorescence Immunohistochemistry Analysis Platforms are Highly Concordant After Linear Calibration

**Figure 1:**
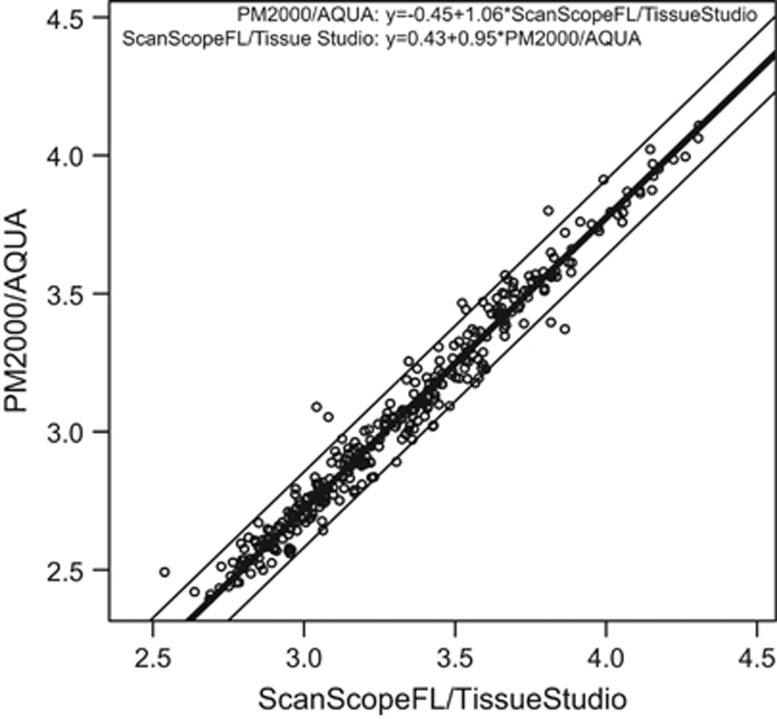
Two technologically distinct platforms for fluorescent image capture and quantitative analysis produce highly concordant quantitative immunohistochemistry values. Quantitative fluorescence immunohistochemistry for nuclear pYStat5 was performed on a cohort of 323 breast cancer patients using two distinct methodologies, PM2000 image capture/AQUA software (PM2000/AQUA) and ScanScopeFL image capture/TissueStudio software (ScanScopeFL/TissueStudio). Log-transformed fluorescence immunohistochemistry values from each platform are displayed by scatter plot. Assay comparison analysis subsequently was performed to generate linear calibration equations from the Bland–Altman difference *vs* mean regression. The thick line corresponds to the conversion equation from Tissue Studio to AQUA log-transformed values, and the thin lines represent the transformed 95% limits of agreement. The two fluorescence immunohistochemistry methodologies showed excellent concordance following linear calibration.

In the absence of an effective gold standard in quantitative fluorescence immunohistochemistry, new assays need to yield comparable results to be deemed reliable. We therefore compared in side-by-side analysis two different promising fluorescence immunohistochemistry image capture and analysis platforms, the PM2000/AQUA and the ScanScopeFL/TissueStudio systems. We first performed fluorescence immunohistochemistry for nuclear-localized and tyrosine-phosphorylated Stat5a/b (Nuc-pYStat5) on 323 breast cancer specimens represented in tissue microarray format on a single histological slide (Cohort 1) that was coimmunostained for pYStat5 and pancytokeratin, and counterstained with DAPI for nuclear detection. The PM2000/AQUA fluorescence immunohistochemistry platform combines capturing of histological images on the PM2000 microscope objective-based scanner and subsequent image analysis using the AQUA software as we have reported previously.^14, 15, 16, 17, 18^ ScanScopeFL/TissueStudio fluorescence immunohistochemistry platform, representing newer hardware/software technologies that we have recently used in our biomarker studies, captures histological images on the ScanScopeFL line scanner with subsequent image analysis using the Tissue Studio software.^19, 20, 21^ A scatter plot of log-transformed fluorescence immunohistochemistry values derived by the two platforms on Cohort 1 indicated excellent concordance across the entire range of values (Figure 1). To directly compare fluorescence immunohistochemistry values obtained from the two image capture and quantification technologies, we performed extended Bland–Altman assay comparison analysis with linear calibration between platforms^23, 24, 25^ as described in the Materials and Methods section. The assay comparison analysis between the two platforms yielded the following equations for the linear calibration: (1) ScanScopeFL/TissueStudio=0.43+0.95 × PM2000/AQUA to calibrate the ScanScopeFL/TissueStudio data to the PM2000/AQUA platform and (2) PM2000/AQUA=−0.45+1.06 × ScanScopeFL/Tissue Studio to calibrate the PM2000/AQUA data to the ScanScopeFL/TissueStudio platform. After linear calibration, the concordance correlation coefficient was 0.984 (95% confidence interval: 0.981, 0.987) between the PM2000/AQUA data and linearly calibrated ScanScopeFL/TissueStudio data and concordance correlation coefficient=0.984 (95% confidence interval: 0.980, 0.987) between ScanScopeFL/TissueStudio data and linearly calibrated PM2000/AQUA data.

The high degree of concordance between the two imaging platforms after linear calibration was confirmed by the analysis of a second non-overlapping tissue microarray cohort (Cohort 2) independently stained for pYStat5, representing tumors from 382 estrogen receptor-positive breast cancer patients (Supplementary Figure 1). After linear calibration, concordance correlation coefficient=0.989 (95% confidence interval: 0.986, 0.991) between ScanScopeFL/TissueStudio data and linearly calibrated PM2000/AQUA data and concordance correlation coefficient=0.988 (95% confidence interval: 0.986, 0.990) between PM2000/AQUA data and linearly calibrated ScanScopeFL/TissueStudio data.

### Two Quantitative Fluorescence Immunohistochemistry Platforms Yield Excellent Agreement in Clinical Outcome Prediction Based on Both Dichotomized and Continuous Marker Levels

**Figure 2:**
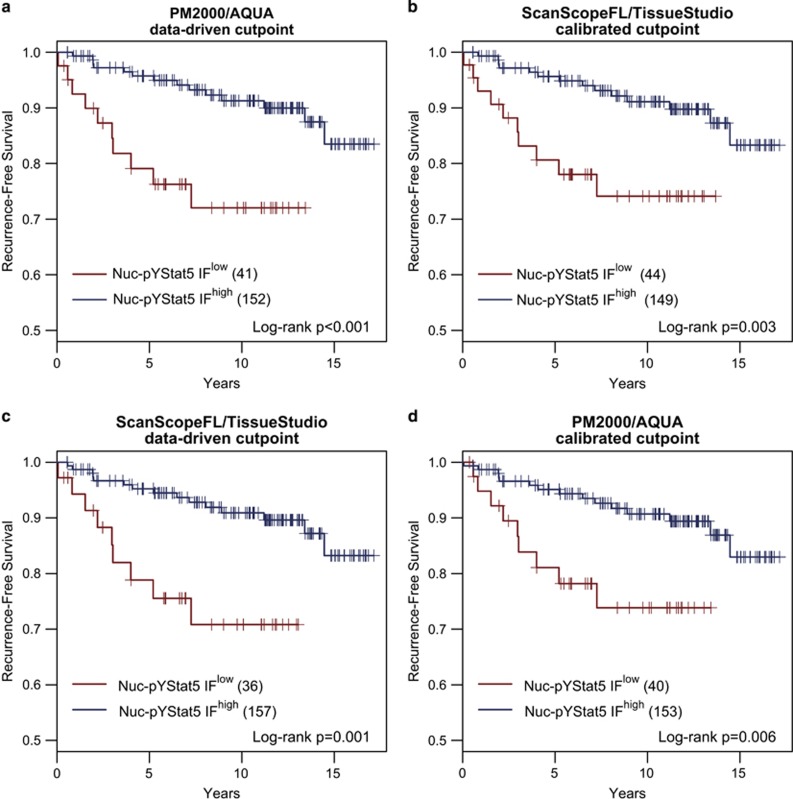
AQUA and Tissue Studio quantification and data-driven dichotomization of nuclear pYStat5 levels reproducibly identify a similar subset of estrogen receptor-positive breast cancer patients with low tumor levels of nuclear pYStat5 at increased risk of disease recurrence. Quantitative fluorescence immunohistochemistry values were computed for Nuc-pYStat5 in 193 estrogen receptor-positive breast cancer specimens using the PM2000/AQUA and ScanScopeFL/TissueStudio platforms. Data-driven, objective cutpoints to identify tumors with low fluorescent immunohistochemistry-detected Nuc-pYStat5 (Nuc-pYStat5 IF^low^) or high Nuc-pYStat5 (Nuc-pYStat5 IF^high^) were derived from data generated on the (**a**) PM2000/AQUA or (**c**) ScanScopeFL/TissueStudio platform, and Kaplan–Meier analysis of recurrence-free survival was performed for each platform. Both platforms identified a similar sub-population of patients whose tumors displayed low Nuc-pYStat5 (≈20%) and were at increased risk of recurrence. The data-driven optimal cutpoint for PM2000/AQUA was linearly calibrated to the (**b**) ScanScopeFL/TissueStudio data using the equation ScanScopeFL/TissueStudio=0.43+0.95 × PM2000/AQUA and the data-driven optimal cutpoint for ScanScopeFL/TissueStudio was linearly calibrated for the (**d**) PM2000-AQUA data using the equation PM2000/AQUA=−0.45+1.06 × ScanScopeFL/TissueStudio. Calibrated cutpoints derived from one platform were then applied to the fluorescence immunohistochemistry values of the alternative platform and subjected to a second Kaplan–Meier analysis (**b** and **d**).

We have previously identified low tumor levels of Nuc-pYStat5 as an indicator of poor prognosis and failure to respond to antiestrogen therapy in estrogen receptor-positive breast cancer patients.^17^ Thus, to further compare the two fluorescence immunohistochemistry methodologies and to determine if data derived from independent platforms yielded similar prognostic utility, we performed survival analysis on the estrogen receptor-positive subset of breast cancer patients from Cohort 1 for whom clinical outcome data was available. The data-driven optimal cutpoints for the PM2000/AQUA platform-derived data revealed a population of estrogen receptor-positive patients, whose tumors constituted the lowest 21% of Nuc-pYStat5-expressing tumors, and who were at a 3.7-fold increased risk of breast cancer recurrence (hazard ratio 3.74 (1.62–8.63), *P*=0.002, *N*=193; Figure 2a). To compare directly clinical outcome predictions between the two assays, we applied the cutpoint derived on Nuc-pYStat5 fluorescence immunohistochemistry values from one assay to the fluorescence immunohistochemistry values derived by the alternative assay. Specifically, we used the linear conversion equations (see Figure 1) to calibrate and apply the cutpoint from PM2000/AQUA to the ScanScopeFL/TissueStudio data, and *vice versa*. The PM2000/AQUA fluorescence immunohistochemistry-derived data-driven optimal cutpoint was linearly calibrated to the ScanScopeFL/TissueStudio data using the PM2000/AQUA conversion equation: ScanScopeFL/TissueStudio=0.43+0.95 × PM2000/AQUA. The resulting cutpoint identified a subset of 23% of patients with low Nuc-pYStat5 as measured by the ScanScopeFL/TissueStudio platform at a 3.3-fold increased risk of breast cancer recurrence (hazard ratio 3.33 (1.44–7.69), *P*=0.005, *N*=193; Figure 2b). The agreement between platforms was also excellent based on the great extent to which the same patients were classified into Nuc-pYStat5-low and Nuc-pYStat5-high groups (Table 1; 95.3% agreement; *κ*-coefficient=0.864 (0.780–0.951); *P*<0.001). The minor fraction of patients (<5%) who were discordantly classified by the two fluorescence immunohistochemistry platforms clustered around the intersect of the data-driven Nuc-pYStat5 cutpoints for the two platforms (Supplementary Figures 2A and B).

Similarly, a data-driven optimized cutpoint for Nuc-pYStat5 derived using the ScanScopeFL/TissueStudio fluorescence immunohistochemistry platform identified a similar population of tumors with low levels of Nuc-pYStat5 (19%) at a comparable 3.7-fold increased risk of breast cancer recurrence (hazard ratio 3.69 (1.57–8.65), *P*=0.003, *N*=193; Figure 2c). After calibrating the ScanScopeFL/TissueStudio optimized cutpoint to the PM2000/AQUA platform-derived data using the ScanScopeFL/TissueStudio conversion equation generated in Figure 1 (PM2000/AQUA=−0.45+1.06 × ScanScopeFL/TissueStudio), the resulting cutpoint for the PM2000/AQUA platform-derived data identified a Nuc-pYStat5-low population of 21% of patients at 3.1-fold increased risk of breast cancer recurrence (hazard ratio 3.12 (1.33–7.27), *P*=0.009, *N*=193; Figure 2d). The ScanScopeFL/TissueStudio-calibrated cutpoint when applied to PM2000/AQUA values also identified highly overlapping sub-populations of patients based on dichotomized Nuc-pYStat5-high and Nuc-pYStat5-low marker status (Table 1 and Supplementary Figure 2B; 95.8% agreement; *κ-*coefficient=0.869 (0.757–0.942); *P*<0.001). Thus, the two quantitative fluorescence immunohistochemistry platforms were highly consistent in identifying clinically relevant sub-populations of patients with low levels of Nuc-pYStat5 at increased risk of breast cancer recurrence using data-driven cutpoints to dichotomize marker levels.

In the present study, Nuc-pYStat5 remained an independent marker of prognosis in estrogen receptor-positive breast cancer based on immunohistochemistry values from either fluorescence immunohistochemistry platform in the multivariable Cox proportional hazards model adjusting for age at diagnosis, tumor grade, nodal status, progesterone receptor status, and Her2 status (data not shown) as observed in the previous patient cohort.^17^ In these models, only progesterone receptor status (negative *vs* positive as determined by clinical chromogen immunohistochemistry score) was a significant predictor of recurrence in addition to Nuc-pYStat5. As the total number of recurrences were relatively low,^24^ only the results from the parsimonious Cox models (reduced to the significant predictors progesterone receptor and Nuc-pYStat5) are reported (Table 2).

We previously have shown using the PM2000/AQUA fluorescence immunohistochemistry platform that low Nuc-pYStat5 is prognostic of poor patient outcome and associated with failure of antiestrogen therapy.^17^ In our previous report, unbiased data-driven cutpoint analysis of PM2000/AQUA-derived Nuc-pYStat5 values identified a subgroup representing 15% of antiestrogen-treated patients whose tumors had the lowest levels of Nuc-pYStat5, with elevated risk of disease recurrence.^17^ We validated this previously established 15th-percentile cutpoint to the corresponding independent subgroup of known antiestrogen-treated patients of Cohort 1, and determined that this previously established cutpoint for Nuc-pYStat5 held up for Nuc-pYStat5 values among the new patients on both the PM2000/AQUA (Supplementary Figure 3A; log-rank *P*=0.003) and ScanScopeFL/TissueStudio (Supplementary Figure 3B; log-rank *P*=0.046) fluorescence immunohistochemistry platforms. The applicability of this previously established cutpoint is of particular importance because the patients were treated at different institutions, with the previously published patient cohort treated at a Canadian institution, whereas Cohort 1 patients in the present study were treated at a US institution.

**Figure 3:**
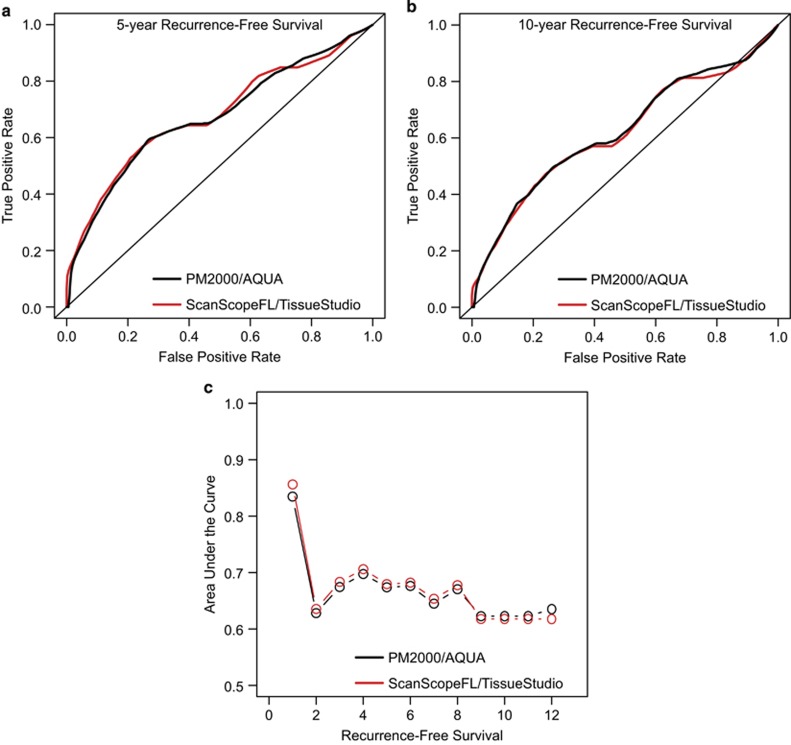
AQUA and Tissue Studio quantification of nuclear pYStat5 levels yielded excellent agreement in clinical outcome prediction based on continuous marker levels. Quantitative fluorescence immunohistochemistry values computed for Nuc-pYStat5 in 193 estrogen receptor-positive breast cancer specimens using the PM2000-AQUA and ScanScopeFL/TissueStudio platforms were used to generate receiver-operating curves for the two platforms for (**a**) 5-year and **b**) 10-year recurrence-free survival. Similar results were observed for corresponding receiver-operator curves computed across all years 1–12 (Supplementary Figure 4), resulting in overlapping area under the receiver-operating curve values (**c**). Notably, this analysis used the raw Nuc-pYStat5 values from each fluorescence immunohistochemistry platform and calibration of the values was not necessary.

In addition to analyzing clinical outcome prediction using data-driven dichotomization of marker levels from the two fluorescence immunohistochemistry platforms, we analyzed Nuc-pYStat5 levels as continuous predictors of clinical outcome. As the clinical outcome is time-dependent, receiver-operating curves were produced for 1-year through 12-year recurrence-free survival using Nuc-pYStat5 levels from each platform. Receiver-operating curves between the two platforms were near identical at both 5- and 10-year recurrence-free survival (Figures 3a and b). Similar results were observed for corresponding receiver-operating curves computed across all years 1–12 (Supplementary Figure 4), resulting in near-identical area under the receiver-operating curve values (Figure 3c). Notably, this analysis used the raw Nuc-pYStat5 values from each fluorescence immunohistochemistry platform and calibration of the values between platforms was not necessary. Collectively, the two quantitative fluorescence immunohistochemistry platforms showed excellent agreement when compared for clinical outcome prediction, regardless of whether we used data-driven dichotomized marker levels or continuous marker levels.

### Quantitative Immunofluorescence Provides Greater Sensitivity and Greater Potential for Classifying Tumors Based on Biomarker Expression Levels

**Figure 4:**
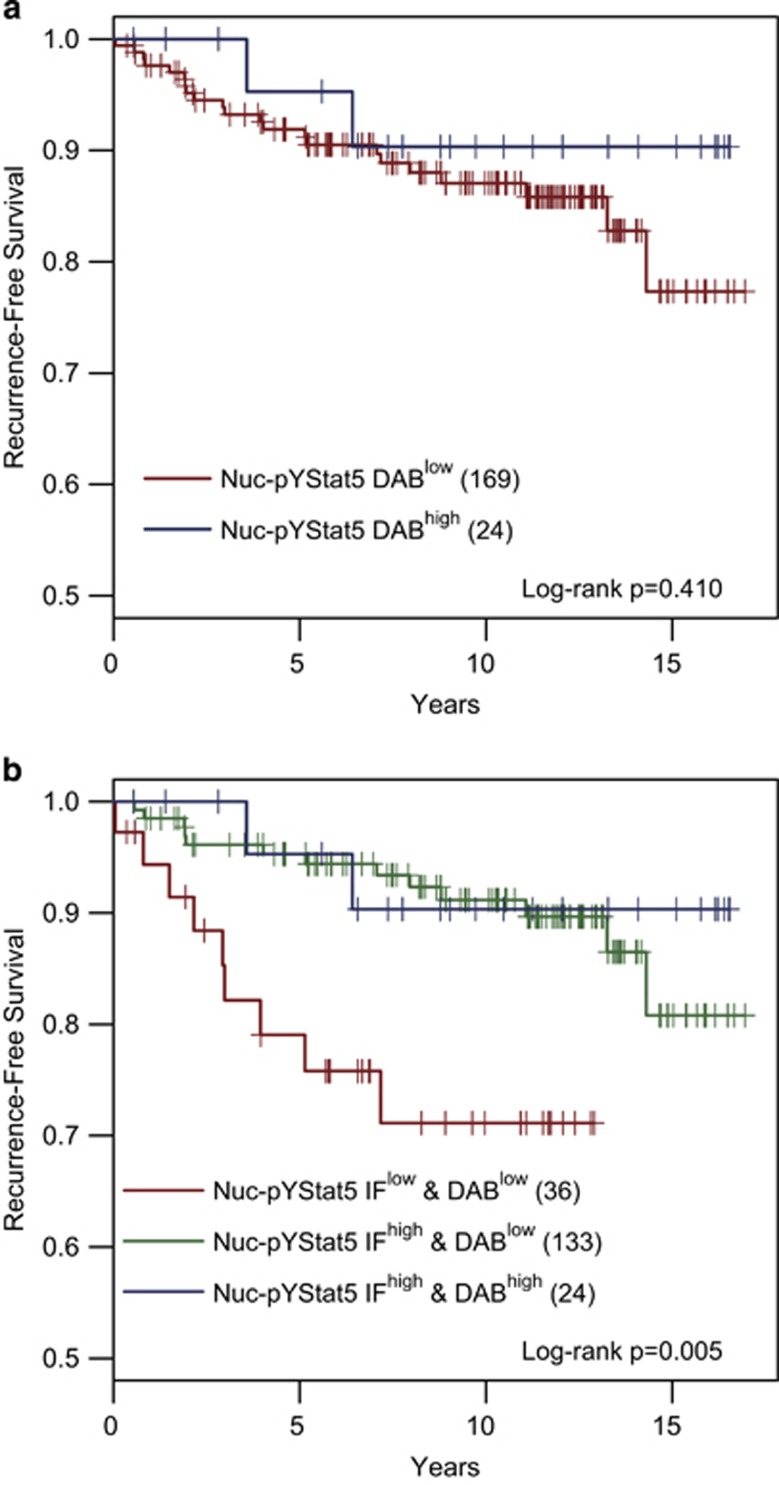
Increased dynamic range of fluorescence immunohistochemistry provides novel information about nuclear pYStat5 expression in breast cancer. Nuclear pYStat5 was evaluated in 193 estrogen receptor-positive breast cancer tumors, comparing standard chromogen immunohistochemistry with pathologist manual scoring and fluorescence immunohistochemistry data. (**a**) Pathologist determination of positive (DAB^high^) and negative (DAB^low^), as determined by ≥1% immunoreactive nuclei, did not readily identify Nuc-pYStat5 as a marker of breast cancer recurrence in Kaplan–Meier analysis of ER-positive breast cancer patients. (**b**) Patients were stratified into three groups based on high/low Nuc-pYStat5 tumor status as determined by fluorescence immunohistochemistry (IF^high^, IF^low^) and positive/negative Nuc-pYStat5 tumor status as determined by chromogen immunohistochemistry (DAB^high^, DAB^low^) and time to breast cancer recurrence was analyzed. Greater resolution and sensitivity of fluorescence immunohistochemistry allowed identification of patients with the lowest Nuc-pYStat5-expressing tumors at elevated risk of recurrence.

Previous studies have found that fluorescence immunohistochemistry by AQUA has greater sensitivity than pathologist-evaluated chromogen immunohistochemistry.^2, 10, 11^ To compare the newer ScanScopeFL/TissueStudio fluorescence immunohistochemistry technology to pathologist-evaluated chromogen immunohistochemistry, we further evaluated the estrogen receptor-positive breast cancers from Cohort 1 for levels of Nuc-pYStat5 using traditional pathologist scoring of chromogen immunohistochemistry. No difference in clinical outcome was observed between the 24 chromogen immunohistochemistry Nuc-pYStat5-positive patients and 169 chromogen immunohistochemistry Nuc-pYStat5-negative patients (Figure 4a). Notably, the chromogen immunohistochemistry Nuc-pYStat5-negative group included the majority of the patients, in contrast to the fluorescence immunohistochemistry Nuc-pYStat5-low group, which represented less than a quarter of the patients (Figures 2a and c), suggesting that reduced sensitivity of chromogen immunohistochemistry impedes identification of the clinically relevant tumors with the lowest levels of Nuc-pYStat5. Consistent with this notion, we observed that the 169 chromogen immunohistochemistry Nuc-pYStat5-negative patients included 36 patients categorized as fluorescence immunohistochemistry Nuc-pYStat5-low and 133 patients categorized as fluorescence immunohistochemistry Nuc-pYStat5-high based on the data-driven optimal cutpoint derived for the ScanScopeFL/TissueStudio platform. In contrast, tumors with high Nuc-pYStat5 levels were readily detected by chromogen immunohistochemistry, as all 24 chromogen immunohistochemistry Nuc-pYStat5-positive patients were also classified as fluorescence immunohistochemistry Nuc-pYStat5-high. Figure 4b shows the Kaplan–Meier progression-free survival curve estimates for the corresponding three groups of patients: (1) fluorescence immunohistochemistry Nuc-pYStat5-low and chromogen immunohistochemistry Nuc-pYStat5-low (Nuc-pYStat5 IF^low^ and DAB^low^, *n*=36), (2) fluorescence immunohistochemistry Nuc-pYStat5-high and chromogen immunohistochemistry Nuc-pYStat5-low (Nuc-pYStat5 IF^high^ and DAB^low^, *n*=133), and (3) fluorescence immunohistochemistry Nuc-pYStat5-high and chromogen immunohistochemistry Nuc-pYStat5-high (Nuc-pYStat5 IF^high^ and DAB^high^, *n*=24). The increased sensitivity and dynamic range of fluorescence immunohistochemistry signals allow further stratification of patients whose tumors were scored negative for Nuc-pYStat5 by chromogen immunohistochemistry and thereby more accurately identifies high-risk patients with low tumor levels of Nuc-pYStat5. These observations support the use of fluorescence instead of chromogen for greater analytic resolution and sensitivity of immunohistochemistry.

### Validation of Operator Variability

**Figure 5:**
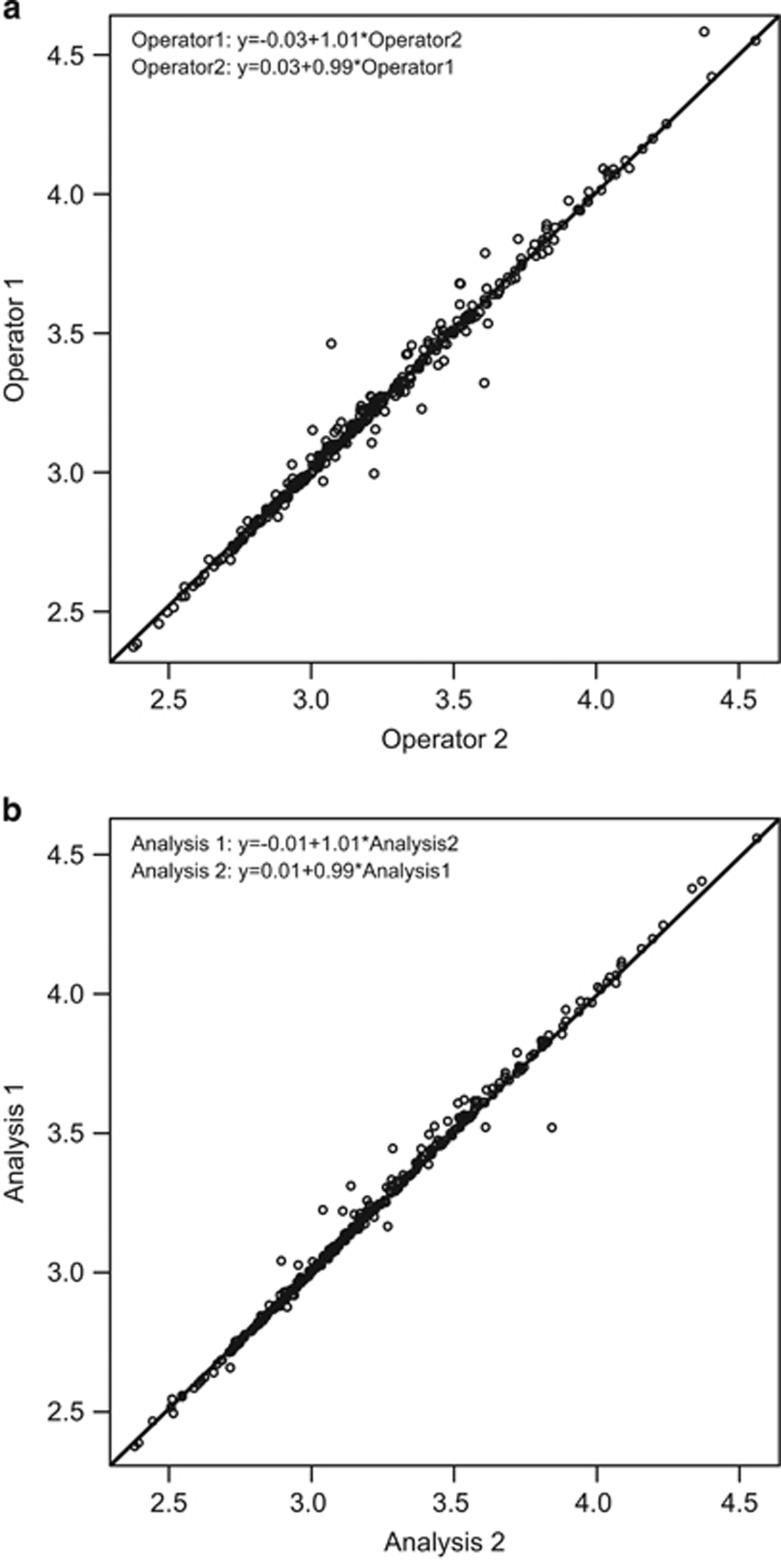
Validation of inter- and intraoperator variability for Tissue Studio analysis of Nuc-pYStat5 in breast cancer by fluorescence immunohistochemistry analysis. (**a**) Two operators (ARP and HR) followed the same standard operating procedure for Tissue Studio image analysis of levels of Nuc-pYStat5 within 336 breast cancer specimens in tissue microarray format, independently selecting tumor tissue features from 12 of the specimens for region-of-interest training according to established guidelines. Interoperator concordance analysis showed high concordance correlation coefficient of 0.993 (95% confidence interval: 0.991, 0.994). (**b**) Intraoperator concordance analysis showed high concordance correlation coefficient of 0.996 (95% confidence interval: 0.995, 0.997) on repeated analysis by the same operator (ARP) using Tissue Studio image analysis. Solid line indicates the line of perfect agreement.

High degree of interoperator concordance has been established previously for the automated PM2000/AQUA platform run under standard operating procedures.^31^ Similar to the AQUA image analysis software, detection of nuclei and cancer cells by Tissue Studio in our fluorescence immunohistochemistry assay is facilitated by DAPI staining of cell nuclei and pancytokeratin staining for epithelial cells and size thresholds. However, unlike the AQUA image analysis software, Tissue Studio analysis includes an operator-guided, machine-learning step performed on a user-selected small subset of representative tissue features to train the software to identify specific regions of interest, in this case cancer cell regions. Because of the potential for subjective or experience-based differences between operators in region-of-interest training, we evaluated the interoperator concordance of Tissue Studio image analysis for the Nuc-pYStat5 fluorescence immunohistochemistry assay. Two operators (ARP and HR) performed independent analyses of 336 breast cancer specimens in tissue microarray format, following an established standard operating procedure for image analysis of levels of Nuc-pYStat5. Each user independently selected examples of cancer and non-cancer within 12 of the 336 tissue cores and performed the analysis following standard protocol. Interoperator concordance was high, with concordance correlation coefficient of 0.993 (95% confidence interval: 0.991, 0.994; Figure 5a). This corresponded to an interoperator coefficient of variation of 1.0% for the Nuc-pYStat5 fluorescence immunohistochemistry image analysis assay. Correspondingly, repeated analysis using Tissue Studio image analysis by the same operator (ARP) also showed high concordance, with concordance correlation coefficient of 0.996 (95% confidence interval: 0.995, 0.997; Figure 5b). This corresponded to an intraoperator coefficient of variation of 0.74%. We conclude that there is low inter- and intraoperator variability for Tissue Studio image analysis of Nuc-pYStat5 levels in multicolor fluorescence immunohistochemistry images.

## Discussion

The present study provides compelling validation of computer-assisted quantitative fluorescence immunohistochemistry for objective measuring of biomarker expression levels in cancer specimens. We identified near-perfect concordance between two independent and technologically distinct quantitative fluorescence immunohistochemistry platforms after linear calibration of fluorescence immunohistochemistry values. The excellent interplatform agreement was based on (1) concordance analysis of immunohistochemistry values derived from two separate analyses of more than 700 breast cancer cases and (2) subsequent clinical outcome analysis. The observed strong agreement between two distinct fluorescence immunohistochemistry image capture and analysis platforms provides direct evidence that objective and valid quantitative data is achievable with commercial fluorescence immunohistochemistry imaging platforms. The two validated platforms provide for the first time benchmarks for further testing of other existing or future fluorescence immunohistochemistry imaging platforms, overcoming the lack of an established gold standard for fluorescence immunohistochemistry methodology.

Fluorescence immunohistochemistry of Nuc-pYStat5 on either imaging platform identified a clinically relevant sub-population of patients with estrogen receptor-positive tumors expressing low levels of Nuc-pYStat5 who were at elevated risk of poor clinical outcome, consistent with previous data on independent breast cancer cohorts from our laboratory and others.^16, 17, 22, 32^ Reflecting excellent concordance between platforms, the data-driven optimized cutpoint derived by AQUA image analysis could, after linear calibration, be applied with excellent agreement to the clinical outcome analyses of the Tissue Studio-derived immunohistochemistry values, and *vice versa*. Furthermore, a data-driven optimized cutpoint for Nuc-pYStat5 fluorescence immunohistochemistry values derived from the PM2000/AQUA platform on a previously reported cohort of antiestrogen-treated breast cancer patients^17^ was independently validated in the present study when applied to the corresponding subgroup of known antiestrogen-treated patients with outcome data using immunohistochemistry values derived from either the PM2000/AQUA or ScanScopeFL/TissueStudio platforms. This progress lends further support to the utility of quantitative fluorescence immunohistochemistry for tumor marker analyses.

Fluorescence immunohistochemistry provides many benefits over standard chromogen immunohistochemistry.^3, 7, 13^ Our data documented the broad dynamic range and sensitivity of fluorescence immunohistochemistry for the Nuc-pYStat5 biomarker when compared with chromogen immunohistochemistry. Fluorescence immunohistochemistry Nuc-pYStat5 values of tumors spanned at least two orders of magnitude on both platforms and linearity held up across the range. Fluorescence immunohistochemistry facilitated identification of patients with extremely low Nuc-pYStat5 who were at markedly elevated risk of breast cancer recurrence, whereas pathologist-evaluated chromogen immunohistochemistry did not readily have sufficient sensitivity to distinguish the lowest Nuc-pYStat5-expressing sub-population of tumors and failed to detect an association with clinical outcome. It is possible that somewhat greater sensitivity could be achieved by improving the pYStat5 chromogen immunohistochemistry assay or by machine-based image analysis of the chromogen-stained tissues. Nonetheless, in addition to greater dynamic range and sensitivity, fluorescence immunohistochemistry provides more effective multiplexing opportunities for parallel quantification of multiple markers than chromogen immunohistochemistry. Additionally, the ability to multiplex staining for cytokeratin and DNA, along with biomarker of interest, facilitates more accurate segmentation of cancer and stromal compartments. Equally important, multiplexed fluorescence immunohistochemistry also offers effective colabeling of regional or cellular structures and thereby facilitates extraction of spatially resolved quantitative information at subcellular, cellular, or tissue compartment levels by imaging software. For instance, in malignant tumors, marker levels can be quantified selectively within cancer cells or in a variety of stromal cells including vascular endothelial cells, leukocytes, or fibroblasts. In the present analysis, we focused on the mean signal intensity of tyrosine-phosphorylated Stat5 within the nuclei of carcinoma cells, a metric that AQUA and Tissue Studio readily compute. Tissue Studio image analysis has additional capabilities, including computation of mean signal intensity within each cancer cell or cancer cell nucleus, and is thus able to supply information about marker distribution across the entire population of cancer cells analyzed. Ongoing work is exploring information benefits embedded in such richer, higher level data. Additional efforts are focused on translating analytical progress on archival tumor tissues in microarray format to clinical biopsies and whole tumor sections.

Objective machine-based tumor marker quantification solutions are expected to greatly enhance pathology practice, but implementation into the clinic has been slow and initially centered on chromogen immunohistochemistry image analyses. Despite the numerous benefits of fluorescence immunohistochemistry, implementation into pathology laboratories has been hampered by limitations in scan speed, extensive data storage requirements, and limitations in computational power. These hurdles are rapidly being lowered through technological advances, and both AQUA and Tissue Studio imaging platforms have been adapted for tumor marker analyses in CLIA-certified central pathology laboratories of Genoptix and Clarient, respectively.^7^ The progress presented in the current study provides further support for the objectivity, sensitivity, and reproducibility of fluorescence immunohistochemistry platforms for clinical tumor biomarker analyses. Furthermore, multiplexed signal quantification at the cellular and subcellular levels, offered by Tissue Studio and other imaging systems, are expected to lead to new predictive companion diagnostic tests that are not achievable by visual assessment. The highly concordant data obtained by two technologically distinct image analysis platforms in the present study support the concept of objective and accurate computer-assisted fluorescence immunohistochemistry analyses of tumor markers. Such platforms are expected to greatly enhance our efforts to characterize drug target expression in cancer and improve personalized cancer therapies.

## Figures and Tables

**Table 1:** Cross-tabulation of estrogen receptor-positive breast cancer patients classified as Nuc-pYStat5-low and Nuc-pYStat5-high by platform-specific data-driven cutpoints and linearly calibrated cutpoints

	*TS: AQUA-calibrated cutpoint*		*Total*
	*Low*	*High*	
*AQUA: data-driven cutpoint*			
Low	38	3	41
High	6	146	152
Total	44	149	193
*κ* (95% CI)=0.864 (0.780–0.951); *P*<0.001			
			
	*TS: data-driven cutpoint*		*Total*
	*Low*	*High*	
*AQUA: TS-calibrated cutpoint*			
Low	34	6	40
High	2	151	157
Total	36	157	193
*κ* (95% CI)=0.869 (0.757–0.942); *P*<0.001			

Abbreviations: CI, 95% confidence interval; TS, TissueStudio.

**Table 2:** Multivariate analysis of Nuc-pYStat5 using PM2000/AQUA and ScanScopeFL/TissueStudio immunofluorescence immunohistochemistry platforms

*Breast cancer recurrence*	*PM2000/AQUA*			*ScanScopeFL/TissueStudio*		
	N	*Hazard ratio (95% CI)*	P*-value*	N	*Hazard ratio (95% CI)*	P*-value*
*Progesterone receptor status*						
Neg	31	4.55 (1.59, 12.50)	—	31	2.73 (1.08, 7.14)	—
Pos	157	1	0.005	157	1	0.034
						
*Nuc-pYStat5*						
Low	42	6.55 (2.41, 17.79)	0.002	38	4.63 (1.91, 11.21)	0.001
High	146	1	—	150	1	—

Abbreviation: CI, confidence interval.
