# Supplementary figures and images for: Validation of tumor protein marker quantification by two independent automated immunofluorescence image analysis platforms
Source: Mod Pathol. 2016 Jun 17;29(10):1143–54. doi: 10.1038/modpathol.2016.112 (PMC5047958; doi:10.1038/modpathol.2016.112)

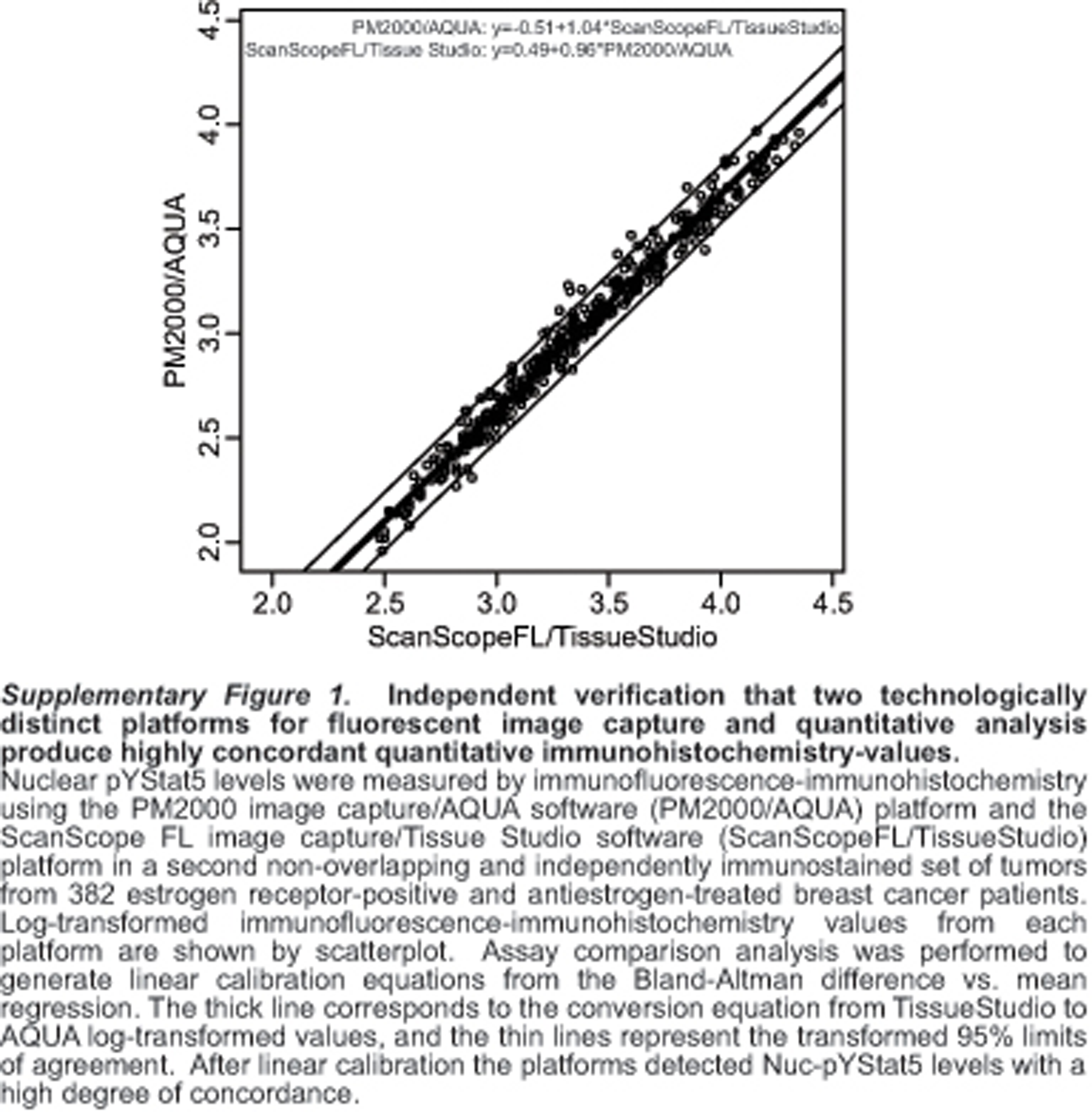

Supplement: Supplementary Figure S1 [file modpathol2016112x1.tif]

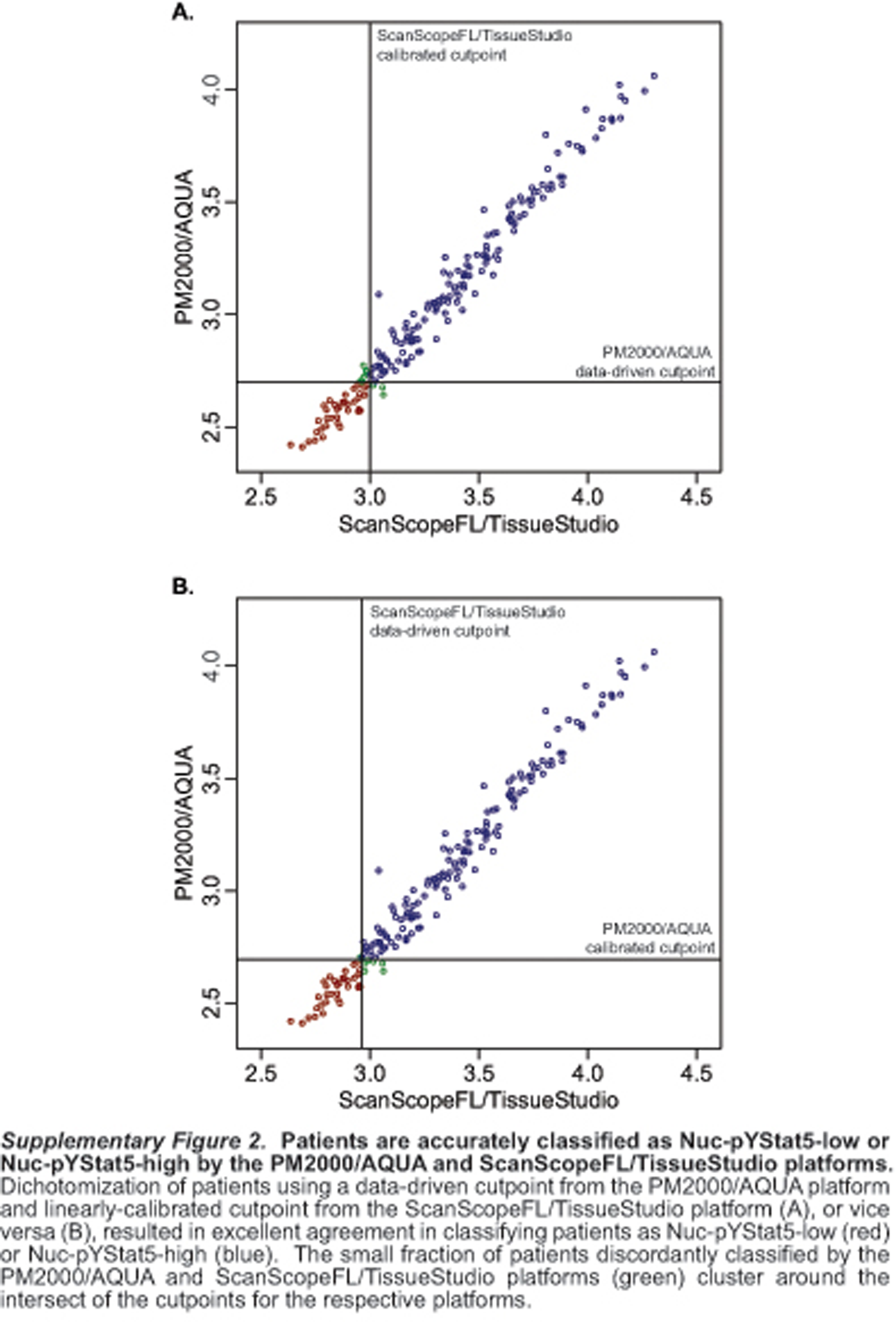

Supplement: Supplementary Figure S2 [file modpathol2016112x2.tif]

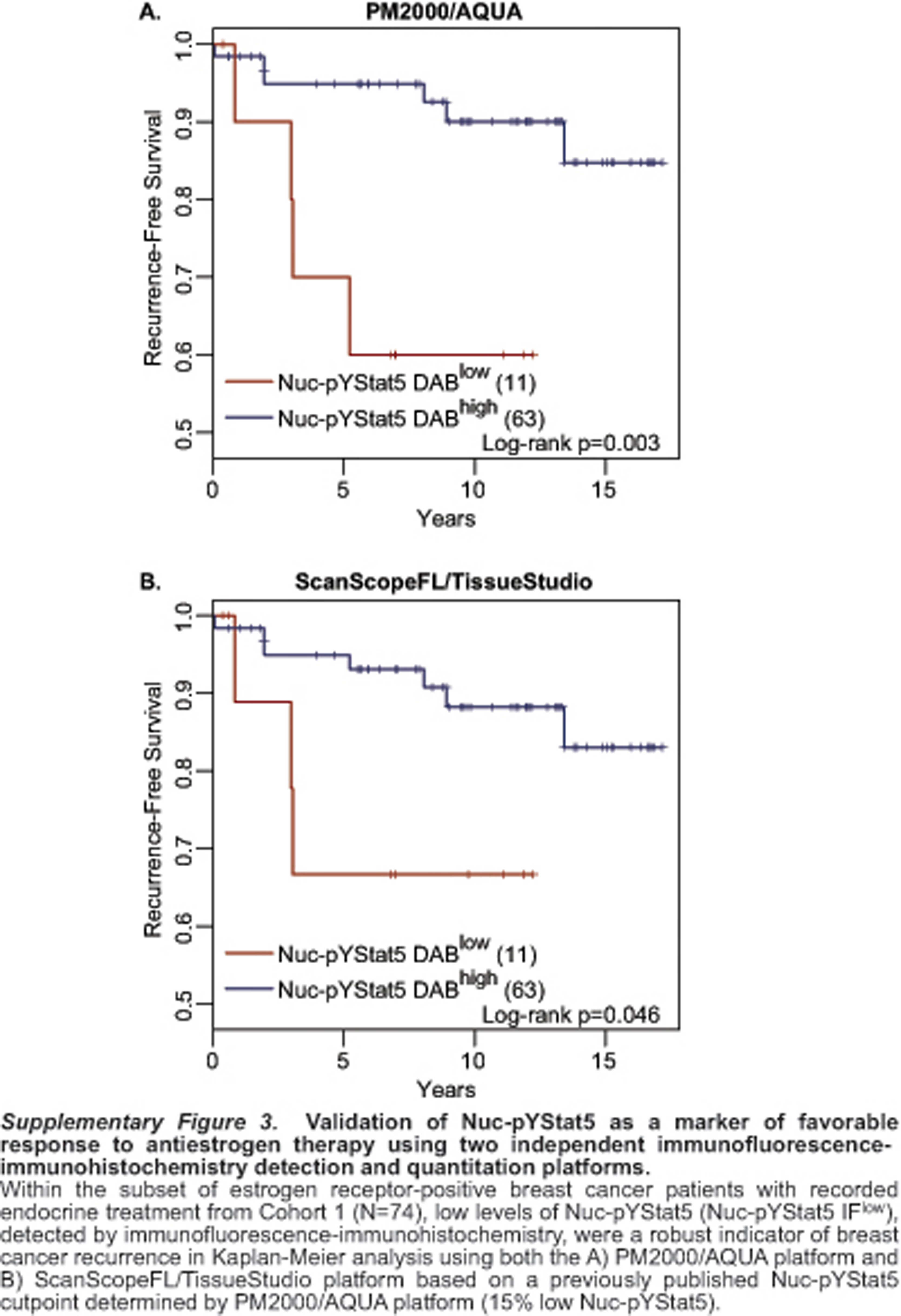

Supplement: Supplementary Figure S3 [file modpathol2016112x3.tif]

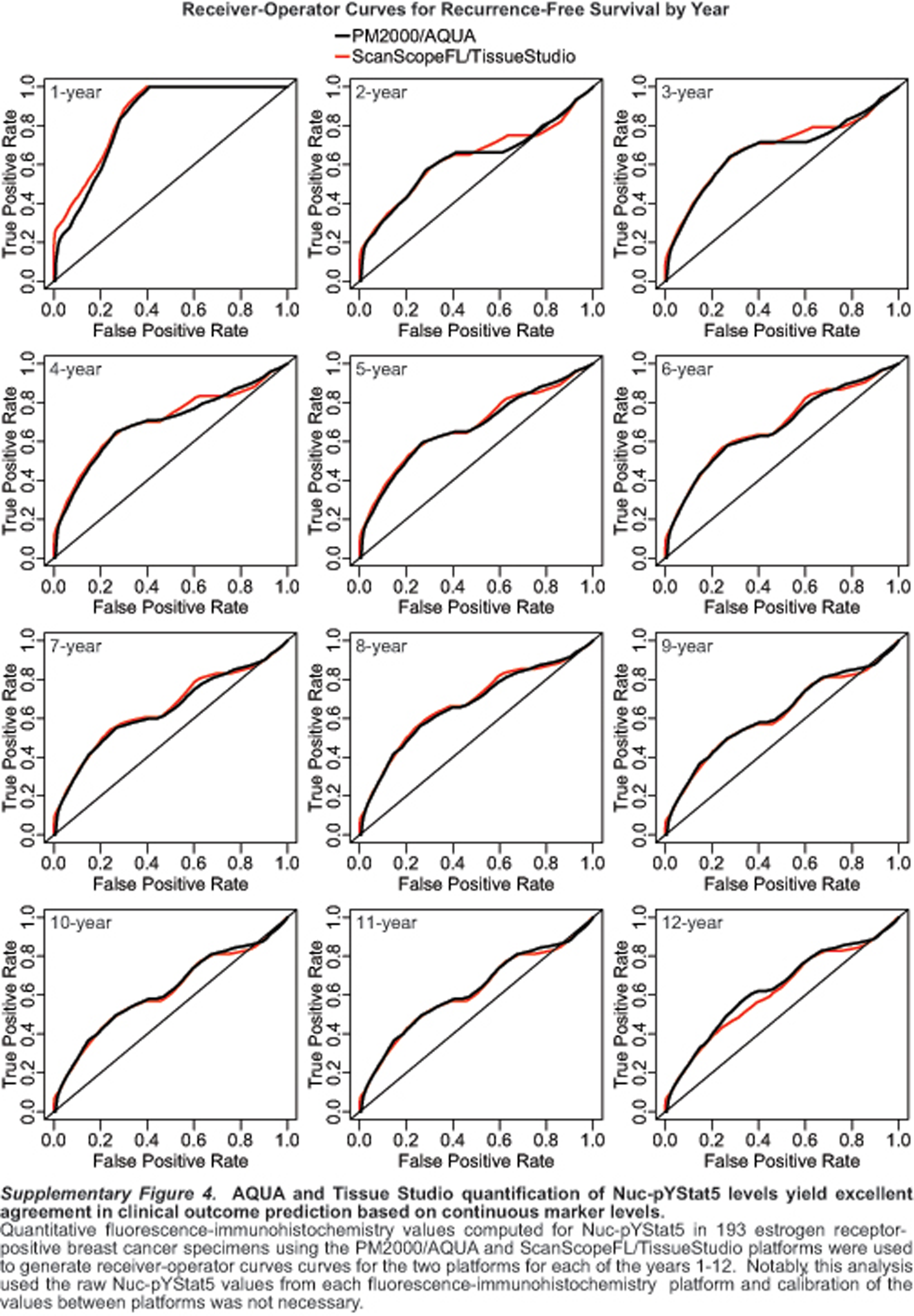

Supplement: Supplementary Figure S4 [file modpathol2016112x4.tif]
